# Evaluation of procalcitonin elevation during ICU stay and its relationship with mortality in ICU patients for COVID-19 with respiratory involvement. A multicenter prospective cohort study

**DOI:** 10.3389/fmed.2022.972659

**Published:** 2022-12-15

**Authors:** Ricardo Rivera-Fernandez, Luis Yáguez-Mateos, María Guerrero-Marin, Rosa María Pérez-Manrique, María Rojas-Amezcua, Antonio Jesús Pontes-Moreno, Juan José Ríos-Toro, Rosa Vela-Colmenero, María Isabel Ruiz-Garcia, Crispín Colmenero-Aguilar, Ana Castillo-Rivera, María Dolores Pola-Gallego de Guzmán, Eduardo Aguilar-Alonso

**Affiliations:** ^1^Intensive Care Unit, University Hospital of Jaén, Jaén, Spain; ^2^Intensive Care Unit, Hospital of Montilla, Córdoba, Spain; ^3^Program in Clinical Medicine and Public Health, University of Granada, Granada, Spain; ^4^Intensive Care Unit, Infanta Margarita Hospital, Córdoba, Spain; ^5^Intensive Care Unit, Hospital San Agustín, Jaén, Spain; ^6^Intensive Care Unit, Hospital of Serrania of Ronda, Málaga, Spain; ^7^Intensive Care Unit, Maimonides Biomedical Research Institute of Córdoba (IMIBIC), Reina Sofía University Hospital, Córdoba, Spain

**Keywords:** procalcitonin, COVID-19, respiratory involvement, ICU, sepsis–diagnostics

## Abstract

**Introduction:**

A multicenter prospective cohort study studied patients admitted to the intensive care unit (ICU) by coronavirus-19 (COVID-19) with respiratory involvement. We observed the number of occasions in which the value of procalcitonin (PCT) was higher than 0.5 ng/ml.

**Objective:**

Evaluation of PCT elevation and influence on mortality in patients admitted to the ICU for COVID-19 with respiratory involvement.

**Measurements and main results:**

We studied 201 patients. On the day of admission, acute physiology and chronic health evaluation (APACHE)-II was 13 (10–16) points. In-hospital mortality was 36.8%. During ICU stay, 104 patients presented 1 or more episodes of PCT elevation and 60 (57.7%) died and 97 patients did not present any episodes of PCT elevation and only 14 (14.4%) died (*p* < 0.001). Multivariable analysis showed that mortality was associated with APACHE-II: [odds ratio (OR): 1.13 (1.04–1.23)], acute kidney injury [OR: 2.21 (1.1–4.42)] and with the presentation of one or more episodes of escalating PCT: [OR: 5.07 (2.44–10.53)]. Of 71 patients who died, 59.2% had an elevated PCT value on the last day, and of the 124 patients who survived, only 3.2% had an elevated PCT value on the last day (*p* < 0.001). On the last day of the ICU stay, the sequential organ failure assessment (SOFA) score of those who died was 9 (6–11) and 1 (0–2) points in survivors (*p* < 0.001). Of the 42 patients who died and in whom PCT was elevated on the last day, 71.4% were considered to have a mainly non-respiratory cause of death.

**Conclusion:**

In patients admitted to the ICU by COVID-19 with respiratory involvement, numerous episodes of PCT elevation are observed, related to mortality. PCT was elevated on the last day in more than half of the patients who died. Serial assessment of procalcitonin in these patients is useful because it alerts to situations of high risk of death. This may be useful in the future to improve the treatment and prognosis of these patients.

## 1 Introduction

The coronavirus-19 (COVID-19) pandemic has affected many patients with high mortality. Many patients have required admission to the intensive care unit (ICU), with mortality very high (1), and knowledge of the prognostic factors of these patients is very important.

Biomarkers are of great help in diagnosis and treatment in different fields of medicine (2–6). Procalcitonin (PCT) is being used as a marker of bacterial infection and to distinguish whether the cause of the clinical picture is bacterial or viral. And as stated by Lippi and Plebani (7), the production and release into circulation of procalcitonin from extra-thyroid sources are greatly amplified during bacterial infections, actively supported by increased concentrations of interleukin (IL)-1β, tumor necrosis factor (TNF)-α, and IL-6. However, the synthesis of this biomarker is inhibited by interferon (INF)-γ, the concentration of which increases during viral infections.

Several meta-analyses and systematic reviews have been performed on the usefulness of PCT for diagnosing bacterial sepsis in critically ill patients (8–10).

Increased PCT values are associated with a nearly 5-fold higher risk of severe SARS-CoV-2 infection (7), and serial procalcitonin measurement may play a role in predicting evolution toward a more severe form of the disease (7, 11). Han et al. (12) postulate that raised PCT observed in COVID-19 could be due either to bacterial co-infection, which is itself causing increased severity and driving systemic sepsis or as a direct marker of a more severe or widespread viral infection. Previous studies (13, 14) find associations between PCT values, severity, and clinical outcomes, especially for mechanical ventilation and all-cause mortality.

Patients admitted to the ICU for COVID-19 with respiratory involvement often require a prolonged stay (15). Patients with prolonged ICU stay present multiple problems that need to be detected and treated. Many studies evaluate the prognostic implications of PCT at a single time point in the evolution of patients with COVID-19 but the prognostic implications at different times during the ICU stay of these patients are less well studied (16). Furthermore, the interpretation of the significance of elevated PCT in ICU patients is difficult to generalize, since these patients frequently develop renal failure (17, 18) and the interpretation of elevated PCT levels is difficult in this group of patients.

We think that it is necessary to investigate further the diagnostic and prognostic implications of elevated PCT values in patients with COVID. We think that the assessment of PCT values at different times of ICU stay may help to increase our knowledge and assist in the management and treatment of these patients with high severity and mortality.

This study aims to analyze PCT levels on admission and during ICU stay in patients admitted to the ICU with respiratory involvement due to COVID-19 and to evaluate its possible prognostic implications.

## 2 Materials and methods

### 2.1 Design

A prospective multicentric cohort study was conducted and we studied all patients admitted to the ICU with respiratory involvement due to COVID-19 in five hospitals. Data were obtained from ICU patients from the following Spanish hospitals in Andalusia: “Hospital Universitario Jaén” in Jaén, “Hospital de San Agustín” in Linares (Jaén), “Hospital de la Serranía de Ronda” in Ronda (Málaga), “Hospital Infanta Margarita” in Cabra (Córdoba), and “Hospital de Montilla” in Montilla (Córdoba). The patients were admitted from 9th March 2020 until August 2021 in one hospital, until November 2020 in two hospitals, and until April 2020 in two other hospitals.”

Coronavirus-19 infection was confirmed in 100% of the patients by a positive result by real-time reverse transcriptase polymerase chain reaction (PCR) for SARS-CoV-2 or detection of IgM or IgG antibodies for SARS-CoV-2, determining infection in the first 15 days and the positivity for IgM.

Admission of patients to the ICU was determined according to the usual criteria of each hospital. In general, in our ICUs the admission criteria were similar, admitting recoverable patients who required specific organic support treatment in the ICU or who required monitoring due to the risk of complications requiring treatment in the ICU. In the specific case of our patients, all of them were affected by COVID-19 with respiratory involvement, the main reason was the need for ventilatory support, with a high percentage of invasive mechanical ventilation.

We collected data on affiliation, previous pathology, and the severity of the process assessed on the first day of admission and the third day with sequential organ failure assessment (SOFA) (19) and acute physiology and chronic health evaluation (APACHE)-II (20).

Procalcitonin values were evaluated daily on certain occasions or in a very high percentage of the days they were in the ICU. A graph of PCT values during each patient’s stay was created for each patient. The number of peaks or occasions during the evolution when the value was in a range above 0.5 ng/ml was observed. Independent peaks in the evolution of the same patient were considered when, after an increase of more than 0.5 ng/ml on one occasion during the evolution, a decrease of at least two-thirds of the maximum value was observed on the following days, followed by another increase (the maximum value of the increase also being greater than 0.5 ng/ml).

Acute kidney injury (AKI) was defined according to AKIN criteria (21): Stage 1–Increase in serum creatinine of more than or equal to 0.3 mg/dl or increase to more than or equal to 150–200% from baseline or diuresis less than 0.5 ml/kg/h for more than 6 h, Stage 2–Increase in serum creatinine to more than 200–300% from baseline or diuresis less than 0.5 ml/kg/h for more than 12 h and Stage 3–Increase in serum creatinine to more than 300% from baseline or creatinine greater than 4 mg/dl with an acute increase of at least 0.5 mg/dl or diuresis less than 0.3 ml/kg/h for 24 h or more or anuria for 12 h or more or the initiation of renal replacement therapy. The plasma creatinine value was measured in all patients on admission to the ICU. However, since many patients are admitted to the ICU with impaired renal function, we also calculated the estimated basal creatinine and considered the basal creatinine to be the lower of the two values.

The estimated basal creatinine value was calculated according to the formula (MDRD), defined as the ideal creatinine value for each patient, assuming a normal glomerular filtration rate of 75 ml/min/1.73 m^2^: (Estimated basal creatinine = (75/[186 × (age^–0.203^) × (0.742 if female) × (1.21 if African-American)])^–0.887^).

For most calculations, the patient was considered to have AKI if they were classified as stage 1, stage 2, or stage 3. The presence of AKI was assessed at different time points where the relationship between increased PCT and mortality was assessed (day 3 of ICU stay, last day of ICU stay, 2 days before ICU discharge, and 5 days before ICU discharge). And for the analysis of the relationship between the presence of 1 or more episodes of elevated PCT during the entire ICU stay, the presence of AKI was considered if at any time the patient presented AKIN criteria of stage 1, stage 2, or stage 3. If the patient presented different stages at different times in his evolution, we classified the patient in the worst stage when it was necessary to use the stage of renal involvement in the statistical analysis.

The treatment of patients conformed to the protocols published by country health authorities and the hospitals participating in the study. Table 1 shows a summary of the therapeutics used by the patients.

**TABLE 1:** Patients characteristic at ICU admission and ICU Interventions.

	Overall (*N* = 201)	Hospital survivors (*N* = 127)	No hospital survivors (*N* = 74)	
Age (years)	63 (56–72)	60 (51–70)	67 (62–74.75)	<0.001
Sex (male)	142 (70.6%)	88 (60.3%)	54 (73%)	0.58
**Medical history**				
Cardiological	114 (56.7%)	69 (54.3%)	45 (60.8%)	0.37
Respiratory	67 (33.5%)	43 (33.3%)	825 (33.8%)	0.95
Kidney	25 (12.4%)	12 (9.4%)	13 (17.6%)	0.93
Liver	5 (2.5%)	1 (0.8%)	4 (5.4%)	0.04
Hematological	23 (11.5%)	10 (7.9%)	13 (17.6%)	0.04
Oncological	22 (10.9%)	12 (9.4%)	10 (13.5%)	0.37
None	39 (19.4%)	28 (22%)	11 (14.9%)	0.21
APACHE II (points)	13 (10–16)	12 (9–14)	15 (12–18)	<0.001
SOFA (points)	5 (3–7)	5 (3–6)	6 (4–8)	<0.005
ICU days	12 (7–26)	11 (7–23)	15 (8–29)	0.09
Mechanical ventilation days (*)	7 (0–17.5)	0 (0–14.5)	19.5 (5–25)	<0.001
P/F ratio *(mm Hg)	145 (111–170)	150 (117–175)	140 (106–160)	0.09
Leukocytes (103/μl)	8,770 (6,390–13,200)	8,410 (5,985–13,055)	9,175 (7,009–13,795)	0.031
Neutrophils 1 day (103/μl)	7,355 (4,535–11,507)	7,055 (4,201–11,449)	7,742 (5,808–11,512)	0.166
Lymphocytes 1 day *(103/μl)	616 (402–957)	622 (406–962)	598 (393–954)	0.61
Lymphocytes 1 day stratification <600	96 (47.8%)	59 (46.5%)	37 (50%)	0.628
Neutrophils 1 day/Lymphocytes 1 day ratio (*)	12.28 (8.05–18.08)	11.56 (7.86–17.1)	13.3 (9.17–19.42)	0.16
Platelets (103/μl)	236 (160–305)	251 (166–320)	210 (151–274)	0.03
Creatinine (mg/dl)	0.91 (0.71–1.16)	0.90 (0.7–1.1)	0.95 (0.8–1.4)	0.03
Urea (mg/dl)	49 (36–67)	45 (34–60)	57 (41–86)	<0.001
LDH (*) (UI/L)	439 (345–593)	411 (320–510)	499 (406–706)	<0.001
AST (*) (UI/L)	41 (29–59)	41 (29–59)	41 (29.7–63)	0.90
ALT (*) (UI/L)	36 (25–61)	37 (25–60.2)	34.5 (24–62.2)	0.53
CK (*) (UI/L)	49 (31–99)	45 (29–85)	56 (41–121)	0.04
CRP (*) (mg/L)	130 (63–204)	123 (63–209)	144 (69–189)	0.97
PCT (ng/mL)	0.14 (0.04–5)	0.10 (0–0.41)	0.26 (0.1–0.5)	0.001
D dimers (*) (μg/L)	1,300 (774–2,321)	1,160 (754–2,023)	1,573 (982–3,815)	0.02
**ICU interventions**				
IPPV	129 (64.2%)	63 (49.6%)	66 (89.2%)	<0.001
Prono	91 (45.3%)	47 (37%)	44 (59.5%)	0.002
Tracheostomy	49 (24.6%)	26 (20.6%)	23 (31.5%)	0.086
corticosteroids	182 (90.5%)	115 (90.6%)	67 (90.5%)	0.998
Hemodiafiltration	18 (9%)	5 (3.9%)	13 (17.6%)	0.01
Vasopressors	122 (66.7%)	59 (46.5%)	63 (85.1%)	<0.001
Empirical antibiotic (*)	165 (82.9%)	99 (78%)	66 (91.7%)	0.14
Antiviral treatment	76 (37.8%)	45 (35.4%)	31 (41.9%)	0.362
Tozilucimab	74 (36.8%)	42 (33.1%)	32 (43.2%)	0.149

*N in those variables is less than the column totals and there are missing values.

### 2.2 Approval of the study by the institutional review board

The study protocol was approved by the Research Ethics Committee of Hospital de Jaén (1508-N-20).

#### 2.2.1 Sample size calculation

For the calculation of the sample size, it was taken into account that the main conclusions would be obtained through multivariate analysis with logistic regression, assuming approximate mortality of 40% and knowing that an approximate number of 10 deceased patients were necessary for each variable included in the multivariable model. We calculated that with a sample of no less than 70 patients it was possible to include a maximum of three variables in the model (22). However, a larger sample would allow us to include more variables in the multivariate analysis if necessary and would increase the reliability of the statistical analysis and the generalizability of the results. For this reason, we will try to obtain a sample of about 200 patients.

### 2.3 Statistical analysis

Continuous variables are expressed as median (25th percentile–75th percentile). Qualitative variables were expressed as absolute and relative frequencies. The Mann–Whitney *U*-test was used for the comparison of continuous variables and the *X*^2^-test for qualitative variables.

Multivariate analysis with multiple logistic regression was performed. Discrimination was assessed with the area under the receiver operating characteristic (ROC) curve and calibration with the Hosmer–Lemeshow test. We also calculated confidence intervals of the odds ratio (OR) by bootstrapping.

The statistical study was carried out with the SPSS and “R” using the “Rcmdr” package and the “Boot” for bootstrapping. We considered *p* < 0.05 as statistically significant.

## 3 Results

We studied 201 patients, APACHE-II was 13 (10–16) points, and the SOFA on the day of admission was 5 (3–7) points. On day 3, 198 patients were alive and SOFA on this day was 5 (3–8) points.

The diagnosis of COVID-19 infection was made by serology in 8 cases and in the remaining cases by PCR. All patients presented respiratory pathology with chest X-ray findings on admission. Mortality was 35.3% in ICU and in-hospital was 36.8% (*N* = 74). ICU stay was 12 (7–26) days.

Table 1 shows the detailed characteristics of patients on admission to the ICU, both those who survived and those who died in the hospital. In-hospital mortality was statistically related to APACHE-II, SOFA, and age.

### 3.1 Analysis of PCT values during the stay in the ICU

During their ICU stay, the 45.3% of the patients did not present any episode of elevated PCT (PCT greater than 0.5 ng/ml), 27.9% presented an only episode and 26.9% presented 2 or more episodes of elevated PCT. The presence of one or more episodes of elevated PCT was statistically associated with mortality, OR: 8.08 (4.07–16.07), (Table 2).

**TABLE 2:** Univariate analysis at different times of the ICU stay.

	Total	Died	Lived	*p*
ICU stay	(*N* = 201)	(*N* = 74)	(*N* = 127)	
≥1 PCT elevation episode (a)	104 (54.7%)	60 (81.04%)	44 (34.6%)	<0.001
APACHE II (points)	13 (10–16)	15 (12–18)	12 (9–14)	<0.001
Acute kidney injury (*n*, %) (b)	100 (49.8%)	52 (73.9%)	48 (37.8%)	<0.001
AKIN stages (c)				<0.001
Stage 0 (*n*, %)	101 (50.2%)	22 (29.7%)	79 (62.2%)	
Stage 1 (*n*, %)	47 (23.4%)	12 (16.2%)	35 (27.6%)	
Stage 2 (*n*, %)	14 (7%)	10 (13.5%)	4 (3.1%)	
Stage 3 (*n*, %)	39 (19.4%)	30 (40.5%)	9 (7.1%)	
AKIN stage 0 during ICU stay	(*N* = 101)	(*N* = 22)	(*N* = 79)	
≥1 PCT elevation episode	36 (35.6%)	13 (59.1%)	9 (13.8%)	0.009
AKIN stage 1 during ICU stay	(*N* = 47)	(*N* = 12)	(*N* = 35)	
≥1 PCT elevation episode	21 (44.7%)	9 (75%)	12 (34.3%)	0.014
AKIN stage 2 during ICU stay	(*N* = 14)	(*N* = 10)	(*N* = 4)	
≥1 PCT elevation episode	12 (85.7%)	10 (100%)	2 (50%)	0.016
AKIN stage 3 during ICU stay	(*N* = 39)	(*N* = 30)	(*N* = 9)	
≥1 PCT elevation episode	35 (89.7%)	28 (93.3%)	7 (77.8%)	0.177
Admission	(*N* = 201)	(*N* = 74)	(*N* = 127)	
PCT elevation at admission (*n*, %)	42 (20.89%)	17 (22.97%)	25 (19.68%)	0.58
SOFA on admission day (points)	5 (3–7)	6 (4–8)	5 (3–6)	<0.005
Admission and SOFA >8 points	(*N* = 38)	(*N* = 22)	(*N* = 16)	
PCT elevation (*n*, %)	15 (39.47%)	8 (36.4%)	7 (43.8%)	0.65
Third-day after admission (d)	(*N* = 187)	(*N* = 68)	(*N* = 119)	
PCT elevation (*n*, %)	45 (24.1%)	26 (38.2%)	19 (*N* = 16%)	0.001
Acute kidney injury in third day (*n*, %)	64 (34.2%)	33 (48.5%)	31 (26.1%)	0.002
SOFA on the third day (points)	5 (3–8)	7 (5–9)	4 (3–6)	<0.001
Third-day after admission and SOFA ≥8 points	(*N* = 50)	(*N* = 29)	(*N* = 21)	
PCT elevation (*n*, %)	17 (34%)	12 (41.4%)	5 (23.8%)	0.196
Third day after admission and acute kidney injury	(*N* = 64)	(*N* = 33)	(*N* = 31)	
PCT elevation (*n*, %)	25 (39.1%)	18 (54.5%)	7 (22.6%)	0.21
SOFA on this day (points)	6 (4–8)	7 (6–9)	4 (3–8)	0.02
Third day after admission and non-acute kidney injury	(*N* = 123)	(*N* = 35)	(*N* = 88)	
PCT elevation (*n*, %)	20 (16.3%)	8 (22.9%)	27 (26.2%)	0.009
SOFA on this day (points)	4 (3–7)	7 (4–8)	3 (2–6)	<0.001
Five days before ICU discharge (e)	(*N* = 172)	(*N* = 65)	(*N* = 107)	
PCT elevation (*n*, %)	39 (22.7%)	29 (44.6%)	10 (9.3%)	<0.001
SOFA on this day (points)	3 (2–6)	6 (4–8)	2 (2–3)	<0.001
Acute kidney injury on this day (*n*, %)	40 (23.3%)	27 (41.5%)	13 (12.4%)	<0.001
Five days before discharge from ICU and acute kidney injury	(*N* = 40)	(*N* = 27)	(*N* = 13)	
PCT elevation (*n*, %)	21 (52.5%)	19 (70.4%)	2 (15.4%)	0.001
SOFA on this day (points)	4 (2–7)	6 (4–9)	2 (1–3)	<0.001
Five days before discharge from ICU and non-acute kidney injury	(*N* = 132)	(*N* = 38)	(*N* = 94)	
PCT elevation (*n*, %)	17 (12.9%)	10 (26.3%)	7 (7.4%)	0.003
SOFA on this day (points)	3 (2–6)	6 (5–8)	2 (2–4)	<0.001
Two days before ICU discharge (f)	(*N* = 197)	(*N* = 72)	(*N* = 125)	
PCT elevation (*n*, %)	41 (20.8%)	34 (47.2%)	7 (5.6%)	<0.001
SOFA points (points)	2 (1–6)	7 (7–9.25)	2 (1–2)	<0.001
Acute kidney injury on this day (*n*, %)	51 (25.9%)	38 (52.7%)	13 (10.4%)	<0.001
Two days before discharge from ICU and acute kidney injury	(*N* = 51)	(*N* = 38)	(*N* = 13)	
PCT elevation (*n*, %)	27 (52.9%)	25 (65.8%)	2 (15.4%)	0.002
SOFA on this day (points)	6 (2–10)	9 (5–10)	18 (1–2)	<0.001
Two days before discharge from ICU and non-acute kidney injury	(*N* = 146)	(*N* = 34)	(*N* = 112)	
PCT elevation (*n*, %)	14 (9.68%)	9 (26.5%)	5 (4.5%)	<0.001
SOFA on this day (points)	2 (1–4)	6 (5–8)	2 (1–2)	<0.001
Last day in ICU (g)	(*N* = 195)	(*N* = 71)	(*N* = 124)	
PCT elevation	46 (23.6%)	42 (59.2%)	4 (3.2%)	<0.001
Acute kidney injury on this day (*n*, %)	54 (27.69%)	45 (36.4%)	9 (7.3%)	<0.001
SOFA on this day (points)	2 (0–7)	9 (6–11)	1 (0–2)	<0.001
Last day in ICU and acute kidney injury	(*N* = 54)	(*N* = 45)	(*N* = 9)	
PCT elevation (*n*, %)	34 (63%)	33 (73.3%)	1 (11.1%)	<0.001
SOFA on this day (points)	9 (1–11)	10 (7–12)	1 (0–1)	<0.001
Last day in ICU and non-acute kidney injury	(*N* = 141)	(*N* = 26)	(*N* = 115)	
PCT elevation (*n*, %)	12 (8.5%)	9 (34.6%)	3 (2.6%)	<0.001
SOFA on this day (points)	1 (0–3)	8 (5–9)	1 (0–2)	<0.001

(a) PCT elevation: PCT greater than 0.5 ng/ml.

(b) Acute kidney injury was considered if the patient was classified as stage 1 or stage 2 or stage 3 of the AKIN classification at any time during the ICU stay.

(c) Stages of acute kidney injury according to AKIN classification during ICU stay.

(d) PCT on the third day was measured in 187 patients (there was one hospital where no patient was evaluated on the third day during the first months).

(e) Data of seven patients were missing and 22 patients were in ICU for less than 5 days.

(f) Data of two patients were missing and two patients were in ICU for less than 2 days.

(g) Data of six patients was missing.

Table 2 shows how mortality is also related to the severity assessed on the first day of admission to the ICU with the APACHE-II and the presence of AKI. Furthermore, the presence of increased PCT is related to mortality both in the total sample and in patients who develop AKI and in those who do not. There is also a statistically significant relationship between increased PCT and mortality in stages 0, 1, and 2 of the AKIN classification.

Multivariate analysis with logistic regression showed that in-hospital mortality was associated with the presentation of one or more episodes of rising PCT during their evolution [OR: 5.07 (2.44–10.53)] as well as with the severity assessed with the APACHE-II and with the presence of AKI (Table 3).

**TABLE 3:** Multivariate logistic regression models at different times of ICU stay.

Model and variables	OR	Confidence OR interval by bootstrapping	ROC area	Hosmer–Lemeshow test
ICU stay			0.81 (0.76–0.87)	7.85 (*p* = 0.45)
APACHE-II	1.13 (1.04–1.23)	(1.03–1.27)		
Acute kidney injury	2.21 (1.10–4.42)	(1.62–5.05)		
>1 episode of elevated PCT	5.07 (2.44–10.53)	(2.44–11.59)		
Third-day after admission			0.80 (0.73–0.86)	12.67 (*p* = 0.124)
Sofa on the third day	1.30 (1.14–1.48)	(1.14–1.51)		
Age	1.08 (1.04–1.11)	(1.04–1.12)		
PCT elevation	2.51 (1.13–5.57)	(1.14–5.87)		
Five days before discharge from ICU			0.92 (0.88–0.96)	12.85 (*p* = 0.117)
SOFA on day 5 before ICU discharge	2.05 (1.64–2.57)	(1.05–2.82)		
Age	1.10 (1.05–1.15)	(1.07–1.17)		
Acute kidney injury	9.73 (3.16–29.98)	(3.89–34.46)		
Two days before discharge from ICU			0.97 (0.95–0.99)	0.893 (*p* = 0.999)
SOFA on day 2 before ICU discharge	3.05 (2.11–4.41)	(2.39–6.23)		
Age	1.16 (1.08–1.25)	(1.09–1.35)		
Acute kidney injury	10.81 (2.21–52.88)	(1.76–137)		
PCT elevation on day 2 before ICU discharge	7.99 (1.32–48.25)	(1.01–169)		
Last day in ICU			0.991 (0.983–0.999)	4.74 (*p* = 0.78)
SOFA on the last day in ICU	2.95 (1.93–4.54)	(2.42–6.23)		
Age	1.18 (1.06–1.31)	(1.10–1.33)		
Acute kidney injury	53.41 (7.28–392)	(2.12–169)		
PCT elevation on the last day in ICU	38.83 (4.27–352.91)	(1.13–159.17)		

### 3.2 Relationship between PCT elevation at admission and mortality

On admission, 42 patients (20.9%) had PCT elevation and 17 (40.5%) died vs. 57 (35.8%) of the 159 patients who did not have PCT elevation (*p* = 0.58).

Subsequently, a similar analysis was performed, but restricted to patients with high SOFA values (≥8 points) on the first day of admission (*n* = 38). In these 38 patients, there was not statistically significant relationship between PCT elevation and mortality (Table 2). PCT was elevated in 15 patients and 8 of them died. The eight patients who died did so after an ICU stay of 7.5 (4–24) days.

### 3.3 Relationship between PCT elevation at day 3 of admission and mortality

On day 3 of admission, PCT was measured in 187 patients (there was one hospital where no patients were assessed on day 3 during the first months). 45 patients (24.1%) had an elevated PCT. Of the 45 patients, 23 of them also had elevated PCT on the day of admission.

Of the 45 patients with elevated PCT, death occurred in 26 (57.8%) compared to 42 (29.6%) of the 142 patients who did not have elevated PCT (*p* = 0.001). Mortality was also related to the severity assessed with SOFA and to the presence of *AKI* on that day (Table 2).

Acute kidney injury was present in 64 patients whose PCT was assessed on day 3. When the relationship of mortality with both variables was studied jointly with multiple logistic regression, we found that it was statistically related to both PCT elevation at day 3 [OR: 2.70 (1.32–5.53)] and the presence of *AKI* [OR: 2.20 (1.15–4.*24*)].

Multivariate analysis with logistic regression showed that in-hospital mortality was associated with SOFA on day 3, age, and elevated PCT on day 3 [OR: 2.51 (1.13–5*.57*)], (Table 3). The presence of *AKI* on that day was not part of the model due to a lack of statistical significance (*p* = 0.26).

Subsequently, an analysis was performed but restricted to patients with high SOFA values (>8 points) on the third day of admission to the ICU (*n* = 50). In these 50 patients, there was not statistically significant relationship between elevated PCT and mortality (Table 2). PCT was elevated in 17 patients and 12 of them died. Furthermore, the 12 patients who died did so after an ICU stay of 18 (5–43) days.

### 3.4 Relationship between mortality with elevated PCT and SOFA in the last days of ICU stay

Subsequently, the last days of ICU stay were analyzed, specifically the last day or the day before discharge from ICU (discharge from ICU as alive or deceased), 2 days before and 5 days before that. On many occasions, patients are in the ICU on the last day for only a few hours and in these cases, laboratory tests were not performed on that day but were performed on the previous day. In these cases, we consider the PCT value on the last day to be the PCT value of the previous day.

The PCT value and SOFA score were evaluated in these three periods. A high PCT value was considered to be present on the last day of the stay or the day before (if the value had not been measured on the last day). If the PCT value had not been assessed on the last two days taken (this occurred in six patients), these patients were excluded from the analysis performed on the last day.

In the last 5 days, 56 patients had elevated PCT, and 47 of them died. Cultures were taken in 45 of the 56 patients. In 25 patients the cultures were positive for bacteria, in five for bacteria and fungi, in four for fungi only, and in 11 were negative. None of the nine positive-fungal cultures were positive in blood and all were in urine or respiratory secretions. And in many of the cases the fungal-positive cultures were considered as colonization.

#### 3.4.1 Evaluation of the last day of admission to the ICU

Mortality was statistically related to SOFA on the last day and elevated PCT on that day to the presence of AKI at that time (Table 2). Elevated PCT was statistically related to mortality in both patients with AKI and those without AKI (Table 2).

Multivariate analysis with logistic regression showed that in-hospital mortality was associated with SOFA on the last day, AKI, age and PCT elevation on the last day [OR: 38.83 (4.27–352.91)] (Table 3).

Of the 42 deceased patients with elevated PCT on the last day, cultures were taken in 36 patients and 23 (54.8%) had positive cultures, and all received antibiotics. Inadequate antibiotic treatment was observed in only two cases.

#### 3.4.2 Evaluation two days before ICU discharge

Mortality was statistically related to SOFA 2 days before ICU discharge, the presence of AKI at that time, and elevated PCT on that day, (Table 2).

Multivariable logistic regression analysis showed that mortality was related to SOFA 2 days before ICU discharge, age, AKI, and elevated PCT on that day [OR: 7.99 (1.32–48.25)] (Table 3).

#### 3.4.3 Evaluation five days before ICU discharge

The same analysis was performed 5 days before ICU discharge. Mortality was statistically related to SOFA 5 days before ICU discharge, the presence of a AKI and elevated PCT on that day, (Table 2).

Multivariate logistic regression analysis showed that mortality was related to SOFA on day 5 before ICU discharge, age, and the presence of AKI at that time (Table 3). There was no statistically significant relationship with increased PCT on that day (*p* = 0.07), OR: 2.92 (0.92–9.32).

### 3.5 Relationship between precipitating cause of mortality and elevated PCT in the last few days

Of the 74 patients who died, 71 were assessed for PCT on the last day. Of those 71 patients, in 42 patients the PCT was elevated on the last day, and 71.4% of them (*n* = 30) were considered to have a mainly non-respiratory cause of death. In 12 (28.6%) patients the precipitating cause of death was considered to be mainly respiratory, 23.8% were considered to be mainly septic shock and in 21.4% multi-organ failure.

Of the 71 patients who died and PCT was evaluated on the last day, in 29 patients the PCT was not elevated on the last day, and in 16 of them (55.2%) the cause was considered to be mainly respiratory.

### 3.6 Graphic representation of PCT values during the ICU stay

**FIGURE 1:**
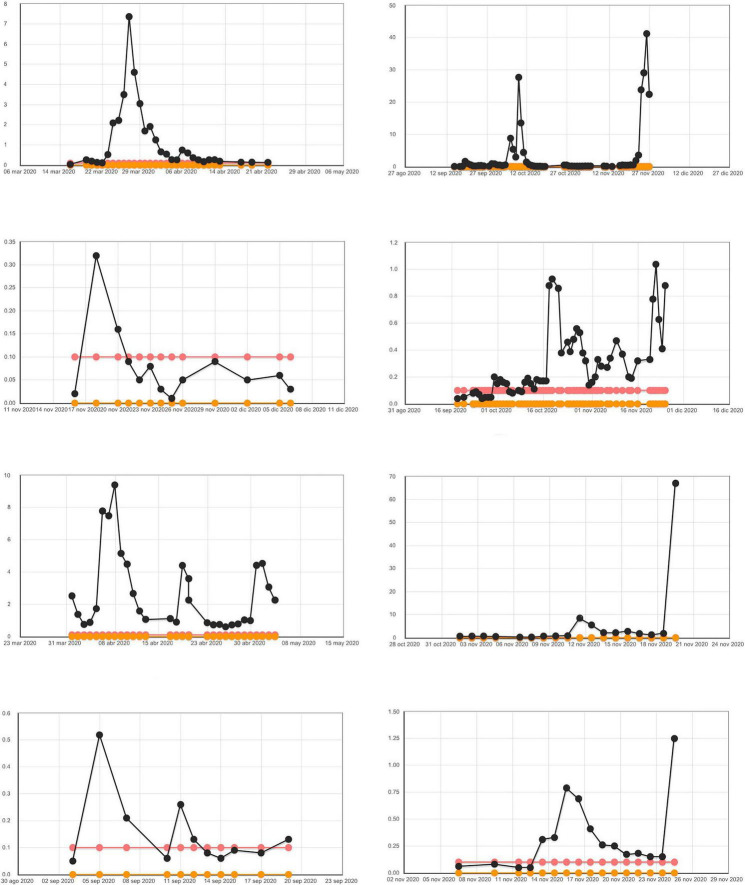
Evolution of PCT during all days of ICU stay in patients who died [lower and upper normal range of laboratory (0–0.05 ng/ml)]. We have considered the value of PCT was higher than 0.5 ng/ml as clinical significance.

**FIGURE 2:**
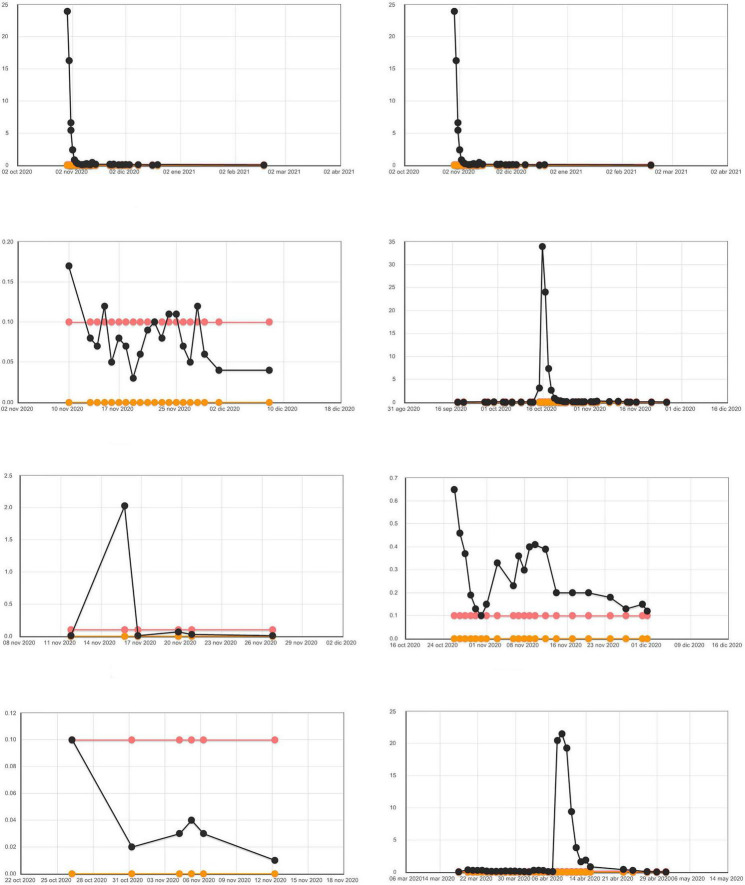
Evolution of PCT during all days of ICU stay in patients who lived [lower and upper normal range of laboratory (0–0.05 ng/ml)]. We have considered the value of PCT higher than 0.5 ng/ml as clinical significance.

**FIGURE 3:**
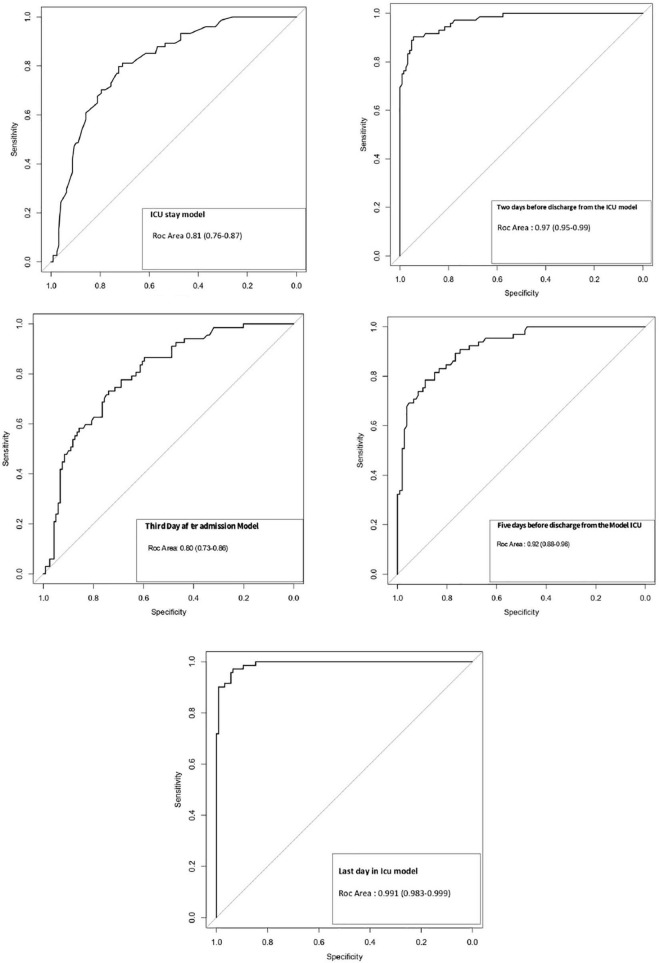
Receiver operating characteristic (ROC) area of multiple logistic regression models.

Figures 1, 2 graphically shows the evolution of PCT during all days of ICU stay in some of the patients of those in whom it was performed. Of note is the absence of graphs from one of the participating hospitals (63 patients). Figure 3 shows the ROC area of multiple logistic regression models about the evolution of PCT during all days of ICU stay of the patients.

These graphs show very clearly that the episodes of elevation of PCT were very persistent during the stay of patients in the ICU, both in deceased patients and in survivors. They also graphically show that episodes of PCT elevation are more frequent in the deceased than in the survivors. And we can also see how many of the patients who die present elevated PCT at the time of death.

## 4 Discussion

In this multi-center cohort study, we serially evaluated PCT values during ICU stay in patients admitted to the ICU for respiratory involvement due to COVID-19 and we studied the relationship of increased PCT values with mortality at different times of ICU stay.

The most striking findings of our study show that patients present numerous episodes of PCT elevation during their stay in the ICU and that they are related to mortality. And our study also shows that these elevations are a warning sign because many of the patients who die have elevated PCT values on the last day and that PCT elevations in the last days of ICU stay are statistically related to mortality. Similar episodes have been observed in many patients on admission and the third day and patients survive in many cases.

It highlights the numerous episodes of elevated PCT levels found, and that even in a quarter of the patients they occur on several occasions. Moreover, the presence of these episodes is statistically associated with mortality. Elevated PCT (7, 11) is associated with severe disease (defined as needing admission to an ICU or use of mechanical ventilation). The patients included in our study can be considered patients with the severe disease since they were admitted to the ICU and a high percentage required mechanical ventilation. Huang et al. (23) found that elevated PCT was associated with increased mortality [RR: 6.26 (1.75–22.42)], which coincides with our results in which we detected an association between mortality and one or more episodes of elevated PCT in patients with severe disease.

Our study also analyses the relationship between mortality and PCT elevation at various times during ICU stay. PCT elevation on the first day of ICU admission was not statistically related to mortality. This relationship was not statistically significant both in the total patients and in the subgroup of patients with the presence of multi-organ failure or with high SOFA scores. If our study had been limited to the analysis of the first day, we could have drawn erroneous conclusions, such as that elevated PCT in patients with COVID-19 is not associated with increased mortality. Possibly the use of prophylactic antibiotic treatment on admission to the ICU in a very large percentage of patients (82.9%) may explain why there is no relationship with mortality between PCT elevation on admission and mortality in our study. However, serial analysis of PCT values during the entire stay of these patients in the ICU has allowed us to see that the relationship between PCT elevation and mortality is statistically significant on the third day and in the last days of the ICU stay. And the relationship was statistically significant with mortality also in the multivariate analysis. The finding of elevated PCT elevation at these times complements the information on age and SOFA at these times.

Our findings are consistent with other studies such as that of Hu et al. (14) who found that in patients with high PCT values who recovered, PCT values decreased during recovery, but in those who died, serum levels of PCT increased as the disease worsened. They evaluated 95 patients of whom 12 were critically ill and six died. We evaluated a larger group of patients, all our patients are critically ill, and 74 of them died. This larger sample has allowed us to obtain statistically significant results, increase the confidence in the conclusions obtained, and also to be able to explore other different aspects. Not being able to know the time of death of the patient entails an important problem in obtaining practical conclusions about the relationship we found between high PCT values in the last days of stay and mortality. We do not know *a priori* whether we are dealing with one of the frequent episodes of elevated PCT that the patient overcomes or one of the episodes that end with the patient’s death. But it does indicate that we must be alert to an elevated PCT because on many occasions it can lead to the death of the patient.

In-hospital mortality in our patients is high (36.8%) but similar to the mortality found by many authors. Armstrong et al. (1) in a meta-analysis with a very large number of patients found an ICU mortality of 41.6% (34.0–49.7%). The fact that our study is a multicenter study and our findings are compatible with previous studies with similar mortality figures makes us more confident that our results can be generalized and be of help for the management and treatment of other patients with this pathology. Although, logically, further research is needed to be able to draw valid and generalizable conclusions.

Although PCT is used as a marker for bacterial sepsis, there is still considerable doubt whether the elevated PCT levels observed in some patients with COVID-19 are due to a bacterial infection or are a direct marker for a more severe viral infection (12).

The use of detailed graphs of patients’ PCT levels and their grouping according to death or survival, allows the reader a complementary and clearer view of the numerical data provided in our study. This makes it possible to observe the high number of cases with elevated PCT in both the deceased and the survivors. The pattern of evolution of PCT levels seen in our patients is more plausible in that it responds to episodes of bacterial infection that complicate the evolution of patients with COVID and that may occur on several occasions in the same patient.

Our data suggest that elevated PCT (in critically ill patients with multiorgan failure and COVID-19 infection) may indicate the presence of bacterial infection and should be treated as best as possible as it causes death in a high percentage of patients. PCT in many cases is indicative of sepsis of bacterial origin (8–10) and we believe that this interpretation can also be applied to our results and that elevated PCT is often due to bacterial infections. Furthermore, the high frequency with which the evolution of COVID patients admitted to the ICU is complicated by bacterial infection must be taken into account, which in some studies reaches percentages of more than 40% (24). The findings of our study show that in the last 5 days of ICU stay, cultures were taken in 45 of the 56 patients with elevated PCT and the cultures were positive for bacteria in 30 of the 45 patients in whom they were taken. Treatment of bacterial infection with antibiotics to which the causative germ is not sensitive is logically a major problem with very serious consequences. Delaying treatment of bacterial infection in ICU patients is detrimental and greatly increases mortality (25, 26) which is why empirical and early antibiotic treatment of bacterial sepsis forms part of the clinical practice guidelines (27).

The use of detailed graphs of PCT levels also allows us to see the high percentage of patients who die with PCT elevation in the last few days and the rapidity of PCT elevation in the short time before death. We have added to our article the PCT progression curves of a group of patients so that the reader can more clearly evaluate the results.

Procalcitonin levels may be increased in acute renal failure. Although this fact is under discussion, there are articles and meta-analyses (28, 29) that conclude that it is acceptable specificity in diagnosing bacterial infection in patients with renal impairment. However, we stratified the population according to renal function and found that elevated procalcitonin is associated with increased mortality in patients with normal renal function and patients with impaired renal function.

Another problem for a proper analysis of our results lies in the fact that the severe respiratory involvement of the patients may be seen by the attending physicians as the sole cause of the patients’ death. This may be the cause of insufficient detection of aggravating factors that could be corrected and treated with the consequent better evolution of the patient. For this reason, we have tried to detect in our study which patients have died due to a deterioration of their respiratory condition and to see which patient’s death is due to a cause other than respiratory deterioration. It has been observed that in many patients, at the time of death, there is an increase in PCT that the precipitating cause of death in many of these cases was not respiratory and that the precipitating cause of death was compatible with a deterioration of infectious origin. All these findings suggest that if detected and treated, it could sometimes prevent the death of the patient.

In our study, a large group of patients who died had elevated SOFA and elevated PCT levels with no response to therapeutic measures. This indicates that patients with elevated SOFA and high PCT are the highest-risk group to whom we need to pay special attention. One of the findings of our study is that in this group of patients, treatment measures are not generally ineffective with better response to therapy in the first days of the stay in the ICU.

These findings suggest that when the time of multi-organ failure is long, the response to therapeutic measures is lower (30, 31). Another possible cause is a higher frequency of limiting therapeutic measures because they are considered to be somewhat futile.

The results of our study show that PCT elevation is a frequent occurrence and that it helps to detect risk situations and can help to better interpret the patient’s situation and the problems that may arise. And it can help in patient management, as has been seen in other situations (32).

### 4.1 Limitations

The number of patients in our study is not very large though it is sufficient to obtain statistically significant relationships on many occasions. The sample is sufficient to show that in patients admitted to the ICU for respiratory involvement by COVID-19, an increase in PCT is a warning sign that the patient is at risk of death. Our study has a sufficient sample to demonstrate that episodes of increased PCT carry a high risk of death, although on many occasions patients survive this situation.

Our study suggests that PCT elevations are caused by a bacterial infection. We found that in the last 5 days of stay, cultures were positive for bacteria in 53% of the patients who had PCT elevations and in 67% of those who had cultures. We did not find positive cultures for bacteria in all cases of PCT elevation. Although it is not enough to affirm that PCT elevation is caused by a bacterial infection in all cases, we have found sufficient data to support that in many cases it is. Although future research is needed to shed more light on this aspect.

## Data availability statement

The raw data supporting the conclusions of this article will be made available by the authors, without undue reservation.

## Ethics statement

The studies involving human participants were reviewed and approved by Research Ethics Committee of Hospital de Jaén (1508-N-20). The patients/participants provided their written informed consent to participate in this study.

## Author contributions

RR-F and RP-M participated in all phases of the study (study design, data collection, statistical review, manuscript completion, and final review). EA-A participated in the statistical review and manuscript completion and prepared the figures and tables. All authors participated in the contribution of data collection.
